# Role of Metastasis-Related Genes in Cisplatin Chemoresistance in Gastric Cancer

**DOI:** 10.3390/ijms21010254

**Published:** 2019-12-30

**Authors:** Yukiko Nishiguchi, Naohide Oue, Rina Fujiwara-Tani, Takamitsu Sasaki, Hitoshi Ohmori, Shingo Kishi, Shiori Mori, Takuya Mori, Naoya Ikeda, Sohei Matsumoto, Kohei Wakatsuki, Yi Luo, Wataru Yasui, Masayuki Sho, Hiroki Kuniyasu

**Affiliations:** 1 Department of Molecular Pathology, Nara Medical University, 840 Shijo-cho, Kashihara, Nara 634-8521, Japan; yukko10219102@yahoo.co.jp (Y.N.); rina_fuji_comma@naramed-u.ac.jp (R.F.-T.); takamitu@fc4.so-net.ne.jp (T.S.); brahmus73@hotmail.com (H.O.); nmu6429@yahoo.co.jp (S.K.); shi.m.0310@i.softbank.jp (S.M.); pt_mori_t@yahoo.co.jp (T.M.); lynantong@hotmail.com (Y.L.); 2 Department of Surgery, National Cancer Center Hospital East, 6-5-1 Kashiwanoha, Kashiwa, Chiba 277-8577, Japan; 3 Department of Surgery, Nara Medical University, 840 Shijo-cho, Kashihara, Nara 634-8522, Japan; nideda@naramed-u.ac.jp (N.I.); smatsumoto@naramed-u.ac.jp (S.M.); kwakatsuki@naramed-u.ac.jp (K.W.); m-sho@naramed-u.ac.jp (M.S.); 4 Department of Molecular Pathology, Hiroshima University Graduate School, 1-2-3 Kasumi, Hiroshima 734-8551, Japan; naoue@hiroshima-u.ac.jp (N.O.); wyasui@hiroshima-u.ac.jp (W.Y.); 5 Jiangsu Province Key Laboratory of Neuroregeneration, Nantong University, 19 Qixiu Road, Nantong 226001, China

**Keywords:** cisplatin, metastasis, HMGB1, gastric cancer, drug resistance

## Abstract

The role of metastasis-related genes in cisplatin (CDDP) chemoresistance in gastric cancer is poorly understood. Here, we examined the expression of four metastasis-related genes (namely, *c-met*, *HMGB1*, *RegIV*, *PCDHB9*) in 39 cases of gastric cancer treated with neoadjuvant therapy with CDDP or CDDP+5-fluorouracil and evaluated its association with CDDP responsiveness. Comparison of CDDP-sensitive cases with CDDP-resistant cases, the expression of c-met, HMGB1, and PCDHB9 was correlated with CDDP resistance. Among them, the expression of HMGB1 showed the most significant correlation with CDDP resistance in multivariate analysis. Treatment of TMK-1 and MKN74 human gastric cancer cell lines with ethyl pyruvate (EP) or tanshinone IIA (TAN), which are reported to inhibit HMGB1 signaling, showed a 4–5-fold increase in inhibition by CDDP. Treatment with EP or TAN also suppressed the expression of TLR4 and MyD88 in the HMGB1 signal transduction pathway and suppressed the activity of NFκB in both cell lines. These results suggest that the expression of these cancer metastasis-related genes is also related to anticancer drug resistance and that suppression of HMGB1 may be particularly useful for CDDP sensitization.

## 1. Introduction

The multidisciplinary treatment of gastric cancer has improved its prognosis. In this treatment modality, cis-dichlorodiammine platinum (CDDP) plays a central role along with 5-fluorouracil (5-FU) and taxanes in chemotherapy for advanced gastric cancer [1]. Chemotherapy with CDDP and S-1 is recommended as the first-line regimen. Predicting CDDP resistance before chemotherapy would be useful in selecting an appropriate chemotherapy regimen. 

Galluzzi et al. divided the mechanism of CDDP resistance into four types [2]: (1) pre-target resistance (DNA binding process of CDDP), (2) on-target resistance (DNA-CDDP adduct formation), (3) post-target resistance (cell death signal due to DNA damage), and (4) off-target resistance. More specific molecular mechanisms of CDDP resistance that have been reported include (1) inhibition of intracellular CDDP accumulation: increase in efflux, decrease in uptake, and detoxification; (2) enhanced DNA repair mechanisms, including nucleotide excision repair, mismatch repair deficiency, and translesion synthesis increase; (3) inhibition of apoptosis; (4) epithelial–mesenchymal transition activation; and (5) increase in stress response chaperons, autophagy, DNA methylation, and changes in microRNA profile [3]. 

CDDP is likely to be administered in patients with advanced gastric cancer, especially to those who have developed metastases. Some metastasis-related genes are highly expressed in primary tumors in gastric cancer with metastasis. The effect of these genes on CDDP sensitivity is unclear. Therefore, studies on the relationship between the expression of such genes and susceptibility to CDDP are necessary. 

c-Met is a receptor for hepatocyte growth factor and is frequently overexpressed in gastric cancer [4]. Its expression level correlates with the progression and metastasis of gastric cancer. In scirrhous type cancer, *c-Met* gene amplification is frequently observed [5]. In addition, chromosome 7q impairment including the *c-Met* gene locus correlates with peritoneal dissemination [6]. HMGB1 is a ligand for RAGE that is highly expressed in various cancers [7], and its co-expression with RAGE correlates with cancer metastasis [8]. HMGB1 is expressed in all types of cells as a nuclear structural protein; however, cancer cells show RAGE activation by HMGB1, which is made available by active secretion and passive release accompanying cell death [9,10]. RegIV is highly expressed in gastric cancer and causes epithelial growth factor receptor activation and stem cell enhancement [11]. RegIV promotes peritoneal dissemination of gastric cancer and promotes resistance to 5-FU [11,12]. *PCDHB9* is a gene of the procadherin family, and its overexpression decreases cell adhesion and promotes peritoneal dissemination [13].

Elucidating the involvement of metastasis-related genes in drug resistance is important in the treatment of advanced gastric cancer. In this study, we investigated the association of the expression of *c-Met*, *HMGB1*, *RegIV*, and *PCDHB9* with CDDP sensitivity, with an aim to reveal new ways to overcome CDDP resistance.

## 2. Results

### 2.1. Expression of the Four Metastasis-Related Genes by Immunohistochemistry

The expression of the four metastasis-related genes was examined by immunohistochemistry in 39 gastric cancer cases. In all cases, neoadjuvant chemotherapy was administered with CDDP alone or in combination as CDDP+5-FU. As shown in Table 1, comparison between the CDDP-sensitive and -resistant cases showed no significant differences with respect to age, sex, cancer histology, or progression.

**Table 1:** Clinicopathological characteristics and expression of metastasis-related genes in 39 gastric cancer cases

Parameter		Resistant	Sensitive	*P* ^2^
Number	39	18	21	
Grade ^1^		0-Ia	Ib-2	
Sex (M:F)		11:7	12:9	NS
Age (years)		72 (58–91)	75 (56–85)	NS
Neoadjuvant	CDDP alone	9	10	NS
	CDDP+5-FU	9	11	
Histology ^3^	tub1	7	6	NS
	tub2	5	6	
	por1	2	3	
	por2/sig	4	6	
pT ^3^	3	3	3	NS
	4a	11	12	
	4b	4	6	
pN ^3^	0	0	0	NS
	1	7	11	
	2	10	7	
	3	1	3	
pM ^3^	0	9	10	NS
	1	9	11	
pStage ^3^	IIIA	5	5	NS
	IIIB	4	5	
	IV	9	11	
Positive Expression ^4^	c-Met	12 (67%)	7 (33%)	NS
	HMGB1	13 (72%)	4 (19%)	0.0013
	REGIV	11 (61%)	4 (19%)	0.01
	PCDHB9	8 (44%)	2 (10%)	0.025

^1^ Histological grade for effectiveness of treatment; grade 1: 0%, grade 1a: 1–33%, grade 1b: 33–67%, and grade 2: 67–99%, depending on the rate of tumor degeneration, necrosis, and disappearance [14]. ^2^ *p* value was calculated by chi-square test. ^3^ Histological and clinicopathological classifications were according to Japanese Gastric Cancer Classification [14]: tub1, well-differentiated tubular adenocarcinoma; tub2, moderately differentiated tubular adenocarcinoma; por1, solid type poorly differentiated adenocarcinoma; por2, non-solid type poorly differentiated adenocarcinoma. sig, signet-ring cell carcinoma; pT3, tumor invades the subserosa; pT4a, tumor invasion is contiguous to or exposed beyond the serosa; pT4b, tumor invades adjacent structures; pN0, no regional lymph node metastasis; pN1, metastasis in 1–2 regional lymph nodes; pN2, metastasis in 3 to 6 regional lymph nodes; pN3, metastasis in 7 or more regional lymph nodes; M0, no distant metastasis; M1, distant metastasis; pStage IIIA, pT3/pN2 and pT4a/pN1-2; pStage IIIB, pT3/pN3, pT4a/pN3 and pT4b/pN1-2; pStage IV, pTany/pNany/pM1. ^4^ According to the cutoff values designated in Figure 1. NS, not significant.

**Figure 1:**
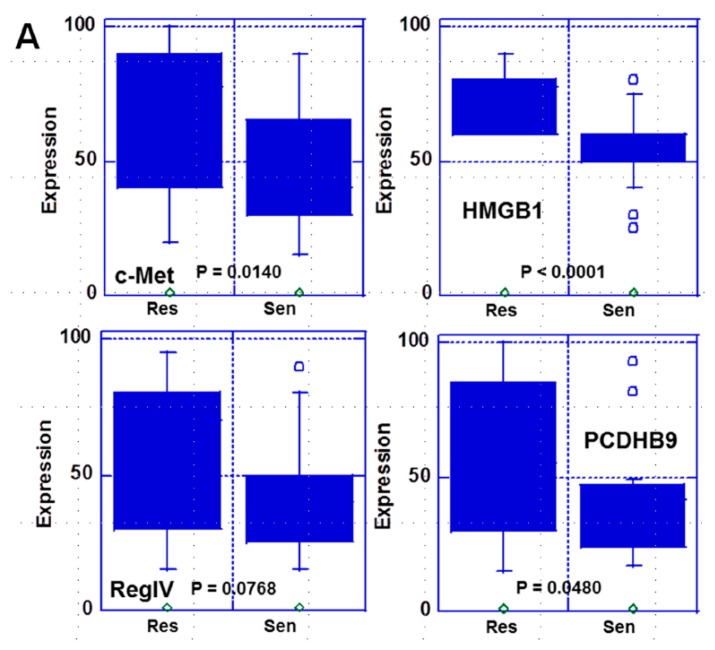

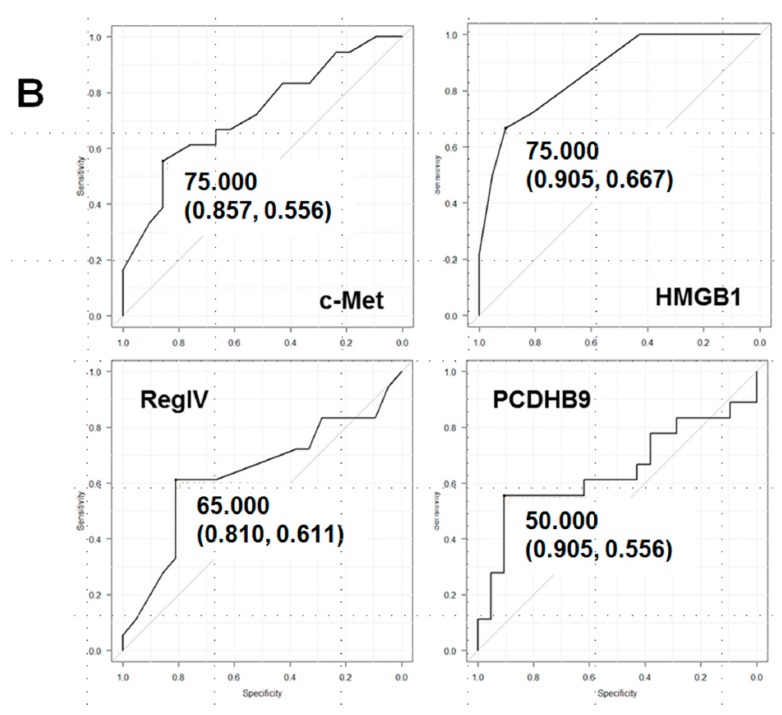
Comparison of expression of the four metastasis-related genes between CDDP-sensitive and CDDP-resistant cases. (**A**) Expressions of the four metastasis-related genes were compared between CDDP-sensitive and CDDP-resistant cases using box-and-whisker plot. Statistical significance was calculated by Student’s *t*-test. (**B**) Receiver operating characteristic (ROC) curve of expression of the four metastasis-related genes on CDDP resistance. Cutoff value (specificity, sensitivity).

**Figure 2:**
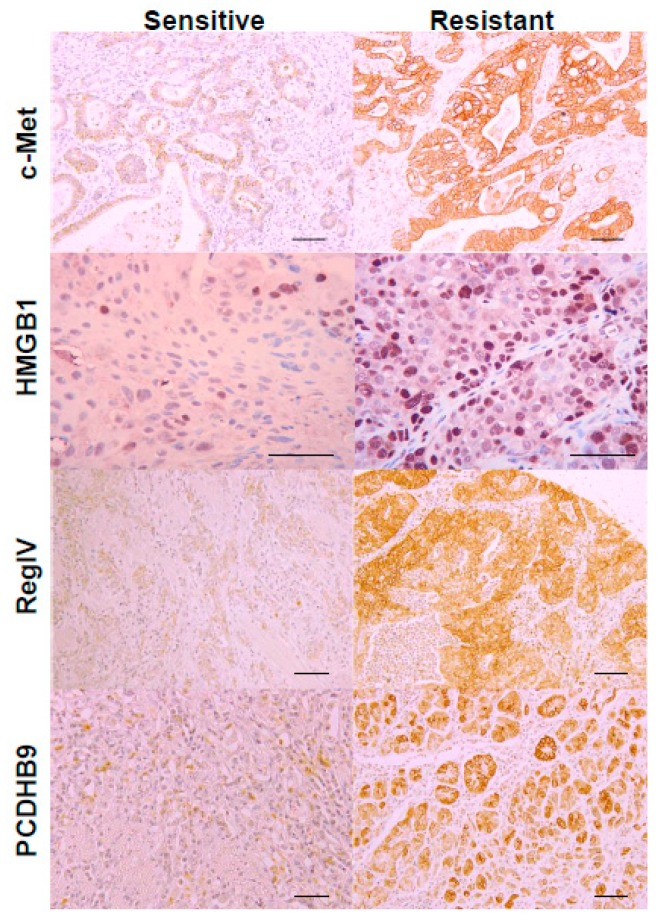
Expression of the four metastasis-related genes in CDDP-sensitive and CDDP-resistant cases. Expression levels of c-Met, HMGB1, RegIV, and PCDHB9 were examined by immunohistochemistry in CDDP-sensitive and -resistant cases. Scale bar, 50 μm.

Upon immunostaining, positive staining was observed in the cytoplasmic membrane and cytoplasm for c-met, PCDHB9 and RegIV, and in the nucleus for HMGB1 (Figure 2). Furthermore, protein expression of all genes was higher in the CDDP-resistant cases than those in the CDDP-sensitive cases.

In 39 gastric cancer cases, the protein expression levels of these genes were compared between sensitive and resistant cases (Figure 1). A significantly higher expression of c-met, HMGB1, and PCDHB9 was observed in resistant cases than in the sensitive cases. Among them, HMGB1 showed the strongest significant difference. In contrast, RegIV expression showed no significant difference. To be able to discriminate the resistant cases from the sensitive cases based on these gene expression levels, the cut-off value for each gene expression was determined by ROC analysis (Figure 1B). The cutoff value of HMGB1 was 75% with a sensitivity of 0.905 and a specificity of 0.667, while c-met had a cutoff value of 75% with a sensitivity of 0.857 and a specificity of 0.556. The number of cases with positive expression was determined using the cutoff value (Table 1). Cases with positive expression of all genes, except c-met, were significantly more frequent among resistant cases than among sensitive cases.

Next, the correlation of these genes with CDDP resistance was examined using multivariate analysis (Table 2). As a result, *HMGB1* showed the strongest correlation among the four genes. We also examined CDDP responsiveness based on the expression of the four genes and using the cut-off values in 10 biopsy specimens obtained before chemotherapy (Table 3). The response to preoperative chemotherapy after biopsy in these cases was sensitive in five cases and resistant in five cases. The true prediction rate for CDDP responsiveness was highest in case of HMGB1 among the four genes (9 out of 10 cases; 90%).

### 2.2. Effect of HMGB1 Inhibition on CDDP Sensitivity in Two Human Gastric Cancer Cell Lines

**Figure 3:**
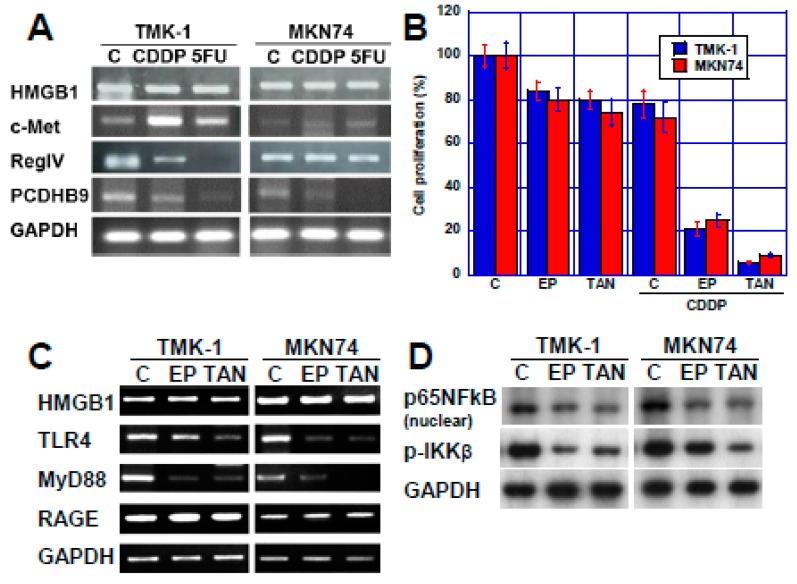
Effect of HMGB1 inhibition on CDDP sensitivity in TMK-1 and MKN74 human gastric cancer cells. (**A**) Expression of the four metastasis-related genes after treatment with CDDP or 5-FU in the two gastric cancer cell lines. (**B**) Effect of CDDP and EP (2 mM) or TAN (20 μM) on cell proliferation. Error bars indicate standard deviation in three independent trials. (**C**) mRNA expression of HMGB1 signaling factors examined in gastric cancer cells treated with EP or TAN by RT-PCR. (**D**) Protein levels of nuclear p65NFκB and phosphorylated IKKβ (p-IKKβ) in gastric cancer cells treated with EP or TAN by immunoblotting. GAPDH served as an internal control. C, control (treated with vehicle DMSO); EP, ethyl pyruvate; TAN, tanshinone IIA.

As described above, *HMGB1* showed the strongest correlation with CDDP responsiveness among the four genes examined this study. Since it was considered that CDDP sensitivity can be promoted by inhibiting HMGB1, we examined the effect of EP and TAN in two types of human gastric cancer cell lines, TMK−1 and MNK74 (Figure 3). First, alteration in the expression of the four genes in the two cell lines treated with CDDP or 5-FU were examined (Figure 3A). The gene expression level of *c-met* increased, while that of *RegIV* and *PCDHB9* decreased upon treatment with anticancer drugs; in contrast, no obvious change was observed in *HMGB1* expression.

Next, we tested the effect of HMGB1 inhibition by treatment with EP and TAN which have been previously reported to inhibit HMGB1, on CDDP sensitivity [15,16,17] (Figure 3B). In both cell lines, growth inhibition rate with CDDP alone was approximately 20%, while it was enhanced to 80% with EP and 10% with TAN. The effect of EP and TAN on the signal transduction system associated with HMGB1 was examined (Figure 3C). Neither EP nor TAN affected the expression of HMGB1 or the expression of HMGB1 receptor RAGE. However, another HMGB1 receptor, TLR4 expression was decreased by EP or TAN. In contrast, the expression of MyD88, a signal transduction factor of HMGB1, was decreased in the two cell lines. Since MyD88 is located upstream of NFκB signaling, the effect of EP and TAN on NFκB signaling was examined (Figure 3D). Upon treatment with EP or TAN, nuclear p65NFkB and phosphorylated IKKβ decreased, which suggested that EP and TAN inhibited NFκB signaling due to inhibiting TLR4-MyD88.

## 3. Discussion

In this study, we investigated the relationship between the expression of four metastasis-related genes in gastric cancer and the sensitivity to CDDP, a major anticancer drug used for treating gastric cancer. As a result, HMGB1, c-met, and PCDHB9 were found to have higher expression in CDDP-resistant cases than that in sensitive cases. This suggests that metastasis and anticancer drug resistance may be due to common gene expression. As a potential cause, emphasis is placed on stem cell promotion and apoptosis suppression common to these genes [18,19].

In our study, the expression level of HMGB1 was most significantly correlated with CDDP resistance among the four genes. HMGB1 is a multifunctional protein that promotes cancer progression and metastasis as a ligand of RAGE or TLR4 in various malignant tumors [7,20,21]. Furthermore, HMGB1 released from cancer cells necrotized by chemotherapy [9] promotes regrowth of the remaining cancer cells and reduces anticancer drug responsiveness [10]. In addition, cytoplasmic HMGB1 promotes cell motility through cdc42 [22,23], and also promotes autophagy and anticancer drug resistance [24]. Nuclear HMGB1 is also involved in DNA repair [25,26], thereby reducing anticancer drug susceptibility and radiosensitivity. In the case of anticancer agents, increased DNA repair capacity results in decreased sensitivity to the drug. In this study, we examined the protein expression level of HMGB1 in the nucleus by immunostaining, which is considered to be appropriate for evaluating the effect of HMGB1 on anticancer drug sensitivity.

Since HMGB1 is thought to bind to CDDP-binding DNA and repair DNA damage, suppressing HMGB1 was thought to reduce the ability of cancer cells to repair DNA and suppress CDDP resistance [26,27]. However, HMGB1 was found to bind to CDDP-bound DNA, thereby inhibiting the access of the repair molecule complex and suppressing the repair [28,29]. Furthermore, HMGB1 shows different CDDP-bound DNA binding properties depending on the four types of molecules generated by post-translational modifications: unmodified (naive HMGB1), oxidized (cysteine oxidation/SS bond formation), acetylated, or phosphorylated HMGB1 [30]. Naive HMGB1 and phosphorylated HMGB1 bind to CDDP-bound DNA and inhibit repair, consequently increasing CDDP sensitivity. In contrast, oxidized HMGB1 and acetylated HMGB1 have reduced binding ability to CDDP-bound DNA and do not cause increased CDDP sensitivity [31,32,33]. Based on these findings, the selective detection of naive HMGB1 and phosphorylated HMGB1 that have high binding ability to CDDP-bound DNA among nuclear HMGB1 can further enhance the specificity and sensitivity of markers for susceptibility of HMGB1 to CDDP.

In the relationship of HMGB1 to tumor stromal cells, cancer-associated fibroblasts (CAF) were reported to induce HMGB1 expression in cancer cells and promote doxorubicin resistance [34]. In addition, CAF-derived HMGB1 promotes TERT expression in cancer cells [35] and induces stem cell properties [36]. Thus, it is considered that HMGB1 promotes anticancer drug resistance by enhancing stemness through the interaction between tumor stromal cells and cancer cells. At this time, HMGB1 is considered to act as a ligand for RAGE or TLR4. Therefore, when we inhibited HMGB1 signaling using EP and TAN, they showed synergistic effects with CDDP. Upon EP or TAN treatment, the expression of TLR4 receptor was decreased and that of myd88 was markedly reduced. Furthermore, downstream NFκB activity decreased. EP is reported to inhibit cancer cell growth and invasion by suppressing HMGB1-RAGE-NFκB axis [15]. TAN is an anti-inflammatory mediator, suppressing HMGB1 signaling [16,17]. Thus, it is suggested that inhibition of HMGB1 as a ligand increases the CDDP sensitivity. Future studies are needed to verify the effect of the specific inhibition of nuclear HMGB1.

Alterations in gene expression associated with anticancer drug treatment might lead to changes in anticancer drug sensitivity. In the four metastasis-related genes examined in this study, expression of RegIV and PCDHB9 was thought to be reduced by CDDP and 5-FU treatment, while c-met expression was found to be increased. This suggests that c-met might be involved in the acquisition of drug resistance associated with anticancer drug treatment. Since the upregulation of *c-met* gene expression by the anticancer drugs was different in the two gastric cancer cell lines, alteration of *c-met* expression is thought to depend on individual tumors. From these findings, it is considered that monitoring the expression of anticancer drug resistance-related genes after starting the administration of anticancer drugs is needed for predicting the acquisition of anticancer drug resistance. In the future, it is expected that the comprehensive reactivity of the expression of metastasis-related genes and the anticancer drug resistance will enable accurate prediction of reactivity to various drugs.

In summary, the expression of cancer metastasis-related genes is suggested to be associated with a decrease in CDDP sensitivity. Particularly, HMGB1 was strongly associated with CDDP resistance. Cancer metastasis and drug resistance are both barriers to cancer treatment and HMGB1 might be a promised molecular target for both in gastric cancer. Trials for HMGB1 targeted therapy should be proactive at the clinical level.

## 4. Materials and Methods

### 4.1. Patients

A total of 39 cases of gastric cancer involving surgical resection with neoadjuvant chemotherapy at the Nara Medical University Hospital and histopathological review by the Department of Molecular Pathology, Nara Medical University, during 2001–2019 were analyzed in this study. As written informed consent was not obtained from patients for their participation in the present study, all identifying information was removed from patient samples prior to their analysis, to ensure strict privacy protection (unlinkable anonymization). All procedures were performed in accordance with the Ethical Guidelines for Human Genome/Gene Research enacted by the Japanese Government and with the approval of the Ethics Committee of Nara Medical University (Approval Number, 937, 1 Apr 2019).

### 4.2. Cells and Reagents

A human gastric cancer cell line, TMK-1, was previously established from a fundic gland-type gastric cancer case [37]. The well-differentiated gastric cancer-derived cell line MKN74 was obtained from the Japanese Collection of Research Bioresources (JCRB; Osaka, Japan). Cells were cultured in Dulbecco’s modified Eagle’s medium (DMEM; Sigma Chemical Co., St. Louis, MO, USA) supplemented with 10% fetal bovine serum (FBS; Sigma). CDDP (5 μg/mL), ethyl pyruvate (EP, 10 μM), and tanshinone IIA (TAN, 2 μg/mL) were purchased from Sigma. All other reagents were of research grade.

### 4.3. Cell Growth and Apoptosis

Cell growth was assessed via a tetrazolium (MTT) dye assay, as previously described [8]. Apoptosis was assessed via the examination of 1000 cells, which were stained with Hoechst 33342 dye (Life Technologies, Carlsbad, CA, USA), and viewed using a fluorescent microscope.

### 4.4. Immunohistochemistry

Consecutive 4-μm sections were immunohistochemically stained using the immunoperoxidase technique described previously [38], with primary antibodies against c-Met (Santa Cruz Biotechnology, Inc., Santa Cruz, CA, USA), HMGB1 (Proteintech Group, Rosemont, IL, USA), RegIV (Biorbyt, St. Louis, MO, USA), and PCDHB8 established in our laboratory [13], and appropriate secondary antibodies (Medical and Biological Laboratories [39], Nagoya, Japan) (all 0.2 µg/mL). The tissue sections were then color-developed with diamine benzidine hydrochloride (DAKO, Glostrup, Denmark) and counterstained with Meyer’s hematoxylin (Sigma). For assessing the expression of c-Met, RegIV, and PCDHB9, cells that exhibited immunoreactivity at the cytoplasmic membrane were counted, and the staining intensity was scored between 0 to 1, (where a score of 0.3 was used to describe the expression level in a normal gastric foveolar epithelium). The staining positivity (0–100) was then calculated as the staining strength score multiplied by the staining area (%). The expression of HMGB1 was assessed by determining the percentage of nuclear immunoreactivity in 1000 examined nuclei.

### 4.5. Reverse Transcription-Polymerase Chain Reaction (RT-PCR)

To assess human CLDN4 mRNA expression, RT-PCR was performed with following cDNA synthesis with 0.5 µg total RNA extracted using an RNeasy kit (Qiagen, Germantown, MD, USA). The primer sets are listed in Table 4 and were synthesized by Sigma Genosys (Ishikari, Japan). PCR products were electrophoresed using a 2% agarose gel and stained with ethidium bromide. The *gapdh* mRNA was also amplified for use as an internal control (NCBI Reference Sequence: BC025925.1).

### 4.6. Statistical Analysis

Statistical significance was calculated using a two-tailed Fisher’s exact test, a one-way ANOVA, and Student’s *t*-test by InStat software (GraphPad, La Jolla, CA, USA). Multiple regression analysis was performed using EZR program [40]. A two-sided *p*-value of < 0.05 was considered to indicate statistical significance.

## Figures and Tables

**Table 2:** Multiple regression analysis of the four metastasis-related genes for CDDP resistance

Genes	Coefficient	95% Interval	Standard Error	*t*-Statistic	*p*-Value
Intercept	−1.072	−1.6362–−0.5089	0.2772	−3.8677	0.00052
*HMGB1*	0.0169	0.0083–0.0255	0.0042	4.0011	0.00035
*c-MET*	0.0011	−0.0042–0.0065	0.0026	0.4308	0.66935
*PCDHB9*	0.0045	−0.0007–0.0010	0.0025	1.7666	0.08627
*REGIV*	0.0034	−0.0019–0.0087	0.0026	1.3162	0.19689

**Table 3:** Prediction of CDDP effectiveness using the four metastasis-related genes

Biopsy No.^1^	Grade ^2^	Effect	*c-Met*	*HMGB1*	*RegIV*	*PCDHB9*
Cutoff ^3^			75	75	65	50
1	1a	Resistant	90 ^4^	95	75	45
2	1a	Resistant	80	90	80	55
3	1a	Resistant	75	90	70	40
4	1a	Resistant	60	90	65	55
5	1a	Resistant	70	60	50	65
6	1b	Effective	60	50	70	55
7	1b	Effective	50	55	75	30
8	2	Effective	50	70	50	35
9	2	Effective	65	70	55	55
10	2	Effective	75	60	50	30
Predictive value			7/10	9/10	7/10	6/10

^1^ Biopsy specimens were obtained before neoadjuvant chemotherapy. ^2^ Histological grade for effectiveness of treatment; grade 1: 0%, grade 1a: 1–33%, grade 1b: 33–67%, and grade 2: 67–99%, depending on the rate of tumor degeneration, necrosis, and disappearance [14]. ^3^ Cutoff values were based on ROC analysis (Figure 1B). ^4^ Positive prediction (i.e., above cutoff value) is emphasized by the underline.

**Table 4:** Primer sequences

Gene	Accession No.	Forward	Reverse
*c-met*	NM_001127500.3	CAGGCAGTGCAGCATGTAGT	GATGATTCCCTCGGTCAGAA
*hmgb1*	CR456863.1	ATATGGCAAAAGCGGACAAG	GCAACATCACCAATGGACAG
*regIV*	NM_001159352.1	TGCTCCTGGATGGTTTTACC	TATCGGCTGGCTTCTCTGAT
*pcdhb9*	NM_019119.4	CACTGAGACAGATGGGCTGA	GCCTTTGTCTTGGAAAGCTG
*tlr4*	AB445638.1	CCTGTCCCTGAACCCTATGA	CCAGAACCAAACGATGGACT
*myd88*	U84408.1	GGATGGTGGTGGTTGTCTCT	AGGATGCTGGGGAACTCTTT
*rage*	AB036432.1	GCTGTCAGCATCAGCATCAT	ATTCAGTTCTGCACGCTCCT
